# Efficacy of Kairomone Lures to Attract Parasitoids of *Halyomorpha halys*

**DOI:** 10.3390/insects14020125

**Published:** 2023-01-25

**Authors:** Kate V. Richardson, Diane G. Alston, Lori R. Spears

**Affiliations:** Department of Biology, Utah State University, Logan, UT 84322, USA

**Keywords:** Scelionidae, *Trissolcus*, Pentatomidae, invasive species, parasitoid, kairomone, chemical ecology, biological control

## Abstract

**Simple Summary:**

The brown marmorated stink bug, *Halyomorpha halys*, is an invasive pest of agricultural crops, ornamentals, and human structures. In its native range, populations are suppressed primarily by parasitoid wasps that attack the egg stage. A promising adventive parasitoid, the samurai wasp, *Trissolcus japonicus*, has become established in the U.S., including Utah. According to ecological models, Utah is marginally suitable for the samurai wasp and poses unique challenges to its establishment from extreme climates and high elevation. Biological control enhancement efforts, such as deploying stink bug kairomones to attract parasitoids, may lead to the enhanced suppression of the brown marmorated stink bug. To evaluate the efficacy of this approach, experimental lures loaded with varying blends and release rates of stink bug kairomones were tested in field and mesocosm trials. This study found low parasitism in the field, while mesocosm trials demonstrated the efficacy of a single- and dual-compound blend at the 10 mg load rate for the attraction of the samurai wasp. These results support the validity of using rubber septa as a release device for kairomones of stink bugs and provide a baseline for future work on attracting samurai wasps with lures in a field environment.

**Abstract:**

In its native range, *Halyomorpha halys* (Stål) is suppressed by parasitoids in the genus *Trissolcus* (Hymenoptera: Scelionidae). *Trissolcus* native to Utah have demonstrated low parasitism of *H. halys*, while adventive *Trissolcus japonicus* (Ashmead) have shown parasitism of up to 20%. Custom rubber septa lures containing stink bug kairomones, n-tridecane (attractant), and (E)-2-decenal (repellent), at 100%, 90%, and 80% levels of attractant (10 mg load rate), were placed adjacent to sentinel *H. halys* egg masses in northern Utah field trials. Egg masses were evaluated for the presence and intensity (proportion of parasitized eggs) of parasitism. Parasitism by *T. japonicus* and *T. euschisti* (Ashmead) was low; however, the 100% lure showed double the parasitism of the control and more than three times that of the 90% and 80%. Two-way choice mesocosm trials in the laboratory evaluated previous lures and a lower load rate of 5 mg—100% attractant treatment. Lures of 10 mg at 100% and 80% were more attractive to *T. japonicus* than the control, while 5 mg at 100% and 10 mg at 90% showed no significant attraction. Our results support a proof-of-concept of rubber septa as release devices for kairomones to attract *T. japonicus* and provide a baseline for future field-based studies.

## 1. Introduction

The brown marmorated stink bug, *Halyomorpha halys* (Stål) (Hemiptera: Pentatomidae), is an invasive pest originating from East Asia. *H. halys* was first detected in Utah in 2012, with the first injury to tree fruit and vegetable crops reported in 2017 [1]. The rapid spread of *H. halys* can be attributed to its ability to utilize more than 300 different host plants including fruits, vegetables, field crops, and ornamentals [2,3,4] upon which it can cause economic damage [5]. In addition to causing mild to severe damage to agricultural crops, *H. halys* is an urban nuisance due to its overwintering congregation behavior on and within human structures [6].

In its native range, *H. halys* populations are suppressed primarily by parasitoid wasps that attack the egg stage [7,8]. The wasp genus *Trissolcus* (Hymenoptera: Scelionidae) contains species that attack *H. halys* eggs; however, those native to Utah have demonstrated only modest parasitism rates of *H. halys* to date [9,10]. *Trissolcus japonicus* (Ashmead), or the samurai wasp, has been identified as a key biocontrol agent with parasitism rates of *H. halys* eggs up to 60–90% in Asia [11]. Adventive populations of *T. japonicus* have been detected in the U.S. since 2014 [12,13], with the first detection in Utah in Salt Lake City in June 2019 [9]. Early surveys found *H. halys* egg masses parasitized by *T. japonicus* on *Catalpa speciosa* (Warder) Warder ex Engelm. (Lamiales: Bignoniaceae) trees [9]; thus, northern catalpa has been a focus for *H. halys* and *T. japonicus* surveys in northern Utah [14].

Utah is a unique geographic location and climate for both *H. halys* and *T. japonicus* with high elevation (>1200 m in northern Utah), substantial snowfall in winter, and hot, arid summers. For up to eight months of the year, average low temperatures fall below the minimum threshold for the development of *T. japonicus* (12.2 °C) [15,16], and current climatic modeling suggests only marginal suitability in the state [17]. A contributing factor may be insect behavioral aspects unaccounted for in laboratory studies and geographic models [17]. Early research in northern Utah indicates *H. halys* egg parasitism rates have increased in the two summers since *T. japonicus* was discovered, thus demonstrating the potential to provide biological control of *H. halys* [9]. Promoting and conserving Utah’s adventive populations of *T. japonicus* may be a viable option for the sustainable management of *H. halys*.

Though lab-reared sentinel *H. halys* egg masses are a common way to assess wasp parasitism rates, many studies have shown that they underestimate parasitism rates and attract fewer wasps than wild egg masses [9,18,19]. It is thought that *T. japonicus* utilizes volatile cues associated with *H. halys* oviposition and feeding during host location. One study found that the bioactive volatile n-tridecane significantly attracted *T. japonicus* and reduced its host search time, while (E)-2-decenal acted as a repellent [20]. Subsequent research by Malek et al. [21] found that combining these kairomone compounds at a ratio of 4:1 n-tridecane to (E)-2-decenal, or 80% attractant, performed better than n-tridecane alone in a Y-tube olfactometer experiment. A more recent study used a ratio of 9:1 (90% attractant) during field trials, but with little success, likely due to inadequate release rates from filter paper sources [22]. The kairomone compounds have proven attractive in small-scale experiments, but more research is needed to understand how they perform in larger settings. 

Traditionally, rubber septa are a standard chemical release device used in pest management (e.g., pheromone lures) [23,24,25]. Septa can be loaded with kairomone compounds to attract beneficial parasitoids and enhance target pest parasitism [26,27]. Our objective was to determine the viability of synthetic kairomone-loaded septa lures to increase *T.japonicus* parasitism rates of *H. halys* egg masses in field and laboratory mesocosm settings. This research will contribute to the identification of effective techniques to deploy stink bug kairomones to attract parasitoids and may contribute to increased accuracy in estimates of *H. halys* parasitism rates and enhance early detection of *T. japonicus*. Future research could lead to the creation of a highly attractive lure for *T. japonicus* with the potential for use in field sites to increase parasitism, thereby reducing *H. halys* populations and subsequent crop and nuisance damage.

## 2. Materials and Methods

### 2.1. Field Trials

To assess the attractiveness of kairomone chemicals in a field setting, custom gray rubber septa lures loaded with 10 mg of test compounds were developed by Trécé, Inc., (Adair, OK, USA). This field study included four lure treatments with varying ratios of n-tridecane to (E)-2-decenal: 100% n-tridecane, 90% n-tridecane, 80% n-tridecane, and hexane (control). Lab-reared *H. halys* sentinel egg masses were deployed adjacent to kairomone lures as hosts for parasitoid wasps.

**Figure 1:**
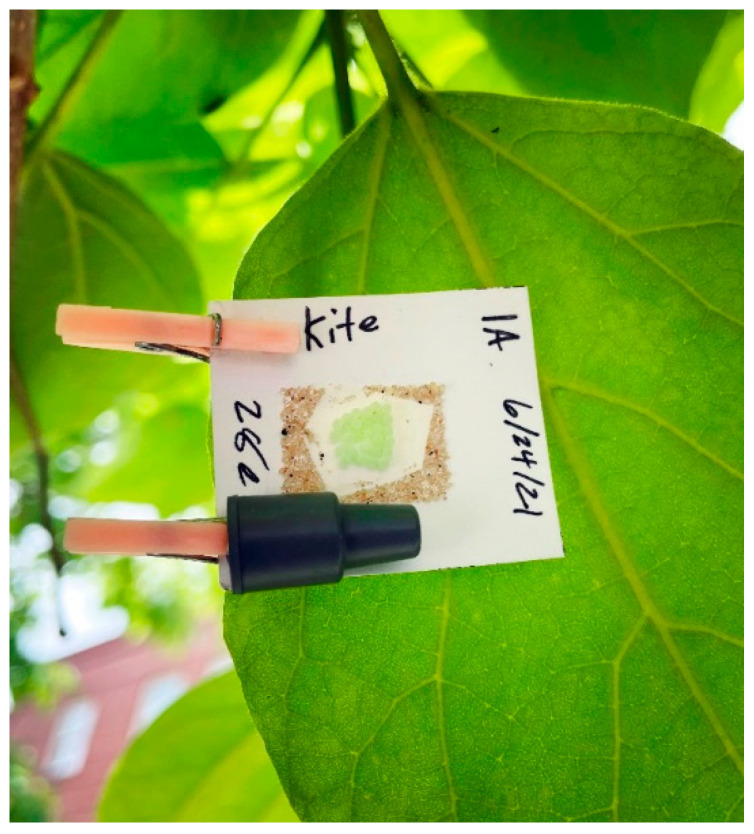
Deployed laboratory reared *Halyomorpha halys* egg mass with adjacent kairomone treatment rubber septa lure on the underside of a northern catalpa leaf in Salt Lake City, UT, from 24 June to 27 August 2021.

Trials (*n* = 6) were conducted from 24 June to 27 August 2021 in a strip of residential *Catalpa speciosa* trees in Salt Lake City, UT (40.772480, −111.854975). Catalpa trees were selected due to consistent and relatively high populations of *H. halys* observed on leaves and pods. Treatments were replicated in four trees (three in the final deployment due to a lack of egg masses) with a blank buffer tree between each treatment tree. Each replicate tree (3 m wide canopy) contained four *H. halys* egg masses and one of each lure treatment with one mass and lure placed approximately 2 m laterally from the trunk of the tree at each cardinal direction. Egg masses of *H. halys* were attached to small rectangles of white cardstock (2 cm by 3 cm) and clipped with a lure to the underside of tree leaves (Figure 1). Treatments were placed at approx. a 2 m height above the ground with cardinal direction of treatments randomized for each tree in each deployment. 

Egg masses (*n* = 92) were ~48 h old and produced from an *H. halys* laboratory colony at the Oregon Department of Agriculture (Salem, OR, USA). Sentinel egg masses were deployed with kairomone treatment lures in the field for approximately 96 h and returned to the laboratory for evaluation. Any wasps found guarding egg masses were collected into a 9-dram plastic vial (Thornton Plastics, Salt Lake City, UT, USA) using a WHO (World Health Organization) in-line tube aspirator (Bioquip, Compton, CA, USA) for later identification. After eggs were incubated at 25–27 °C for 14 days in the laboratory, they were evaluated for parasitism incidence and intensity (proportion of parasitized eggs per mass), and emerged wasps were identified to species using the key to Nearctic *Trissolcus* [28]. Egg masses were observed again 14 days later (4 weeks after collection) to identify late-emerging wasps or eggs with partially developed wasps or stink bugs. Individual egg fate was recorded as emerged or undeveloped parasitoid, hatched or unhatched *H. halys* nymph, predated, sunken, empty, or missing. 

### 2.2. Mesocosm Trials

In order to mitigate multiple uncontrollable factors in a field setting, a second experiment was conducted in a mesocosm-scale lab-based experimental system in 2022. In a 0.5 m height × 0.5 m depth × 0.8 m length plexiglass observation cage with a mesh lid, a clear panel trap (Alpha Scents, Inc., Canby, OR, USA, 30.5 cm × 30.5 cm) and lure attached via a clothespin to the upper portion of the card were hung at either end of the cage (1 m apart). Four to five 1–20 day post-emergence (according to colony availability), honey-fed, female *T. japonicus* from the Utah State University *T. japonicus* colony (originating from females collected in 2019 from Salt Lake City, UT, USA) were introduced to the center, bottom of the cage via a small access hole (stoppered by a cork) and allowed up to 30 min to select between the control and treatment lure by landing on the adjacent clear panel sticky trap (2-way choice). Treatments consisted of four combinations of chemical attractant to repellent ratios and load rates: 5 mg–100% n-tridecane, 10 mg–100% n-tridecane, 10 mg–90% n-tridecane, and 10 mg–80% n-tridecane. 

Each treatment was tested against a hexane control lure and trials were replicated until each treatment had a minimum of 32 individuals make a choice. Wasps that did not make a choice within the 30 min experimental period were not included in the analyses. The observation cage was cleaned with laboratory detergent and allowed to air dry between lure types and treatment vs. control lures were rotated every other trial to account for side bias. Trials were conducted in a temperature-controlled rearing room at 21–27 °C and 30–50% RH.

### 2.3. Statistical Analysis

To analyze field trial results, data were pooled across sample dates and each egg fate category was analyzed using the Kruskal-Wallis test to compare differences across treatment lure types. In mesocosm trials, the null hypothesis that *T. japonicus* showed no preference between the control and treatment lure (an overall choice of 50:50) was analyzed with a Chi-square goodness of fit test. All statistical comparisons were run using R software [29] and were considered significant at *p* < 0.05.

## 3. Results

### 3.1. Field Trials

**Figure 2:**
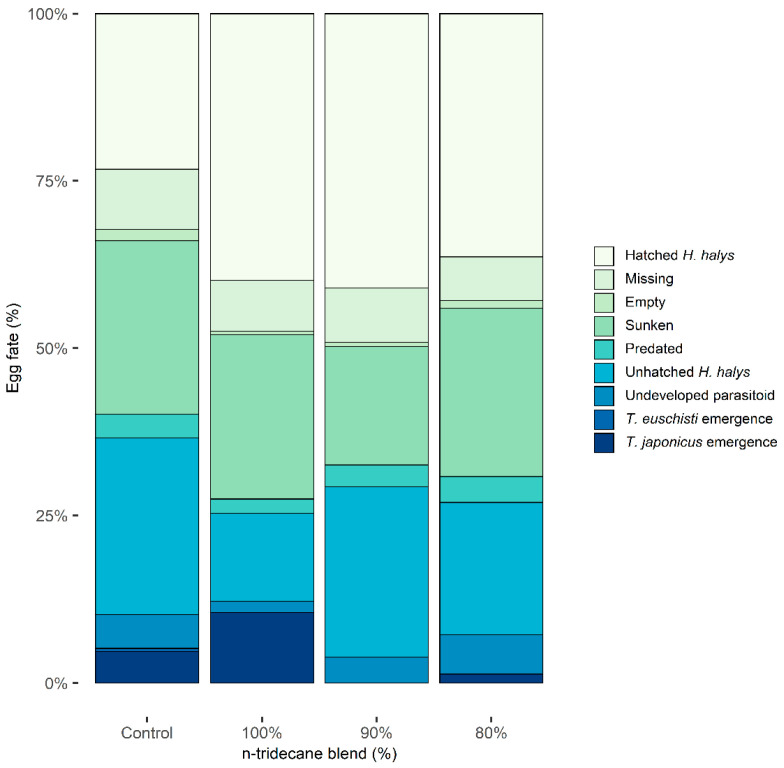
Fate of field-deployed *Halyomorpha halys* eggs (*n* = 2472) in each kairomone lure treatment, shown as percentage of total eggs. Lure treatments are labeled based on percentage of n-tridecane attractant to (E)-2-decenal repellent: hexane (control, *n* = 598), 100% (*n* = 615), 90% (*n* = 617), and 80% (*n* = 597). A total of 6 egg mass deployments were made in Salt Lake City, UT, USA from 24 June through 27 August 2021.

Of 92 total egg masses containing 2472 eggs deployed in the field, only 13 masses had evidence of parasitism (14.1%; guarding female present or eclosed parasitoids) of which 6 masses supported successful emergence of adult parasitoids (6.5%). The lure treatments in order of highest proportion of eggs with successfully emerged parasitoids were 100% n-tridecane (10.6%), hexane control (5.2%), 80% n-tridecane (2.9%), and 90% n-tridecane (0.0%) (Figure 2). Though the 100% lure showed double the parasitism of the control and more than three times that of the 90% and 80%, the analysis of all egg fate categories did not support statistical differences (*p* > 0.05; Supplementary Table S1). 

*T. japonicus* attacked 5.4% of deployed egg masses. This included one egg mass in each of the control and 80% attractant treatments and three in the 100% attractant treatment. Of the 129 *H. halys* eggs *T. japonicus* parasitized, there was a high rate of successful emergence (78.3%). *T. euschisti* attacked 6.5% of egg masses deployed with two egg masses in each of the control, 100%, and 90% attractant treatments and none in the 80% attractant treatment. Successful emergence of *T. euschisti* only occurred in one of the control treatment egg masses and the rate of overall emergence from attacked egg masses (136 eggs) was 1.8%. Two egg masses in the control treatment were parasitized by an unknown *Trissolcus* sp(p) and demonstrated no successful emergence (Table 1). In one instance, a *T. euschisti* female was found guarding an egg mass which later produced 26 *T. japonicus* individuals (92.9% emergence rate) and 2 inviable eggs.

Undeveloped parasitoid rates ranged from 1.6% to 5.9% across treatments, were highest in the 80% attractant treatment, and lowest in the 100% attractant treatment. The rate of unhatched *H. halys* was also variable across treatments, making up the largest percentage of egg fate for the control treatment (26.5%), and was noticeably lower in the 100% attractant treatment compared to others. There was less than a 2% difference in the predation between lure treatments. Sunken rates were relatively high (17.7–26.0%), and the combination of missing and empty on average across lure treatments made up <10% of egg fate. Rates of hatched *H. halys* nymphs were similar across lure treatments containing stink bug kairomones (36.4–41.0%) but were unexpectedly low for the control lure making up approximately half of that seen in non-control lures.

### 3.2. Mesocosm Trials

**Figure 3:**
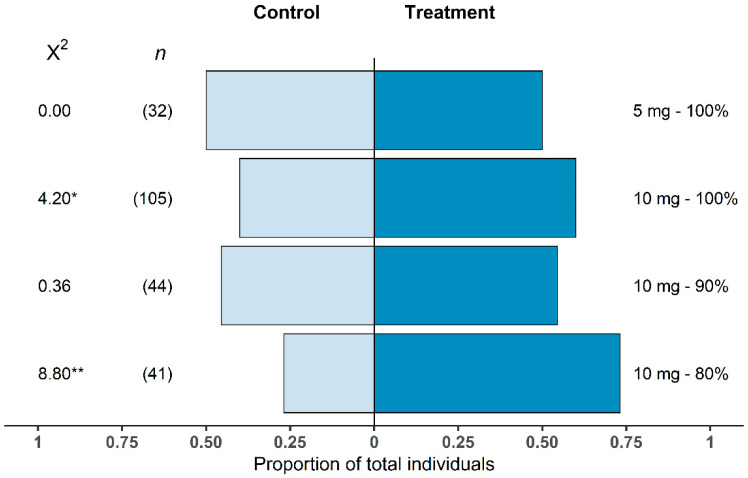
Proportional response of *Trissolcus japonicus* female adults that chose a kairomone lure treatment or control (*n* = 32–105) within the 30 min experimental period. Chi-squared values are presented and numbers in parentheses represent sample size; * *p* < 0.05; ** *p* < 0.01.

In the mesocosm system, *T. japonicus* females showed a significant preference for the kairomone blend over the control for two of the four treatments tested (Figure 3). The 10 mg—80% n-tridecane showed the most significant response (*p* = 0.003) with 73% of individuals choosing the kairomone lure. There was also a significant difference between the control and 10 mg—100% n-tridecane treatment lure (*p* = 0.04) with 60% of wasps choosing the kairomone-treated side. For the 10 mg—90% n-tridecane treatment, 55% of wasps chose the kairomone lure; however, there was no significant deviation from the expected response (*p* = 0.55). The choice between the low load rate treatment of 5 mg—100% n-tridecane and the control also showed no significance (*p* = 1); an even proportion of wasps chose each side. The proportion of no-choice wasps per treatment varied from 20% to 32% of wasps tested and was not associated with treatments.

## 4. Discussion

This study is the first to assess and provide a proof-of-concept for kairomone-infused rubber septa lures to attract *T. japonicus* and other potential *H. halys* parasitoids in field and mesocosm settings. Though the kairomone chemicals tested herein have been previously evaluated in a small-scale lab setting (Y-tube olfactometer) [20], implementation in the field may provide different results due to the influence of external factors on the shape, concentration, longevity, and spatial extent of the kairomone odor plume. In addition, the interaction of the lure plume with nearby plant surfaces and mixing with other volatiles may give rise to plume masking or plume amplification, of which little is known in a parasitoid searching context [30]. 

Our field results contrast those of Malek et al. [21] where the combination of the attractant and repellent was preferred over the attractant alone. In our study, not only did the 100% n-tridecane attractant lure have a numerically (not statistically) higher parasitism rate than the control, the lures containing the previously identified repellent (E)-2-decenal had less parasitism than those without the repellent, including the hexane control. These results may suggest that under field conditions a combination of the attractant and repellent is less effective at increasing parasitism by *Trissolcus* spp., at least with the specific load rate tested and placement in near proximity to the target host egg masses.

The lures used in the field study contained a load rate of 10 mg per lure, high in comparison to standard pheromone lures (1 mg), in the hopes of ensuring the attraction of parasitoids (Trécé, Inc., Adair, OK, USA); however, this relatively high load rate may have been counterproductive to our objective. In studies of pheromone lure load rates, it has been found that attraction often plateaus and can even become repellent to the insect at high release rates [23,24,25]. In addition, our lures were placed directly adjacent to the *H. halys* egg masses (Figure 1), which may deter parasitoid attraction as the host female stink bug does not typically remain near the eggs following oviposition. 

The effect of lure load rate on parasitoid attraction was explored by examining both a 5 and 10 mg treatment of only the attractant n-tridecane in the mesocosm trials. The results of these trials demonstrate that the higher load rate of 10 mg was a more viable option for attracting *T. japonicus* and did not have a repellent effect. In contrast, the 5 mg treatment demonstrated no difference in attraction as compared with the hexane control lure. Based on these findings, the load rate may not have been detrimentally high in the field trials; however, we did not assess if a load rate greater than 10 mg could increase parasitoid attraction in a field setting. 

Given the low number of egg masses tested in field trials and a lack of statistically significant results, the parameters of this field study may have been unsuitable to discern differences in the attractiveness of the different kairomone lure treatments. We observed low parasitism rates across all egg mass deployment periods in this study. In previous surveys, the site selected for the field study had the highest abundance of *T. japonicus* observed in northern Utah with concomitant high parasitism rates of *H. halys* [9]. However, compared to *T. japonicus* populations in its native geographic regions and other adventive populations in the U.S., the abundance of *T. japonicus* in Utah is relatively low [13,31]. In addition, Utah suffered from significant drought over the time frame of this study (summer 2021), with 99.94% of the state in “extreme” or “exceptional” drought categories [15], which may have had a negative effect on host abundance, parasitoid wasp populations, and *H. halys* egg parasitism rates [6].

Other researchers have observed that lab-reared egg masses perform inferiorly to wild egg masses in terms of their attractiveness to parasitoids [9,18,19]. The lures tested here were an initial attempt to solve this issue and increase the accuracy of parasitism rates detected in deployed egg mass surveys. Parasitism was observed in wild *H. halys* egg masses near deployed, unparasitized egg masses during the field study. Without an in-depth analysis of the kairomone plume release from lures, it is unknown if the plumes for each lure treatment may have overlapped within the tree block (lures were separated by approximately 3 m of tree canopy) and potentially interfered with one another. In addition, this may suggest the lure-dispersed kairomones had unknown interactions with the surrounding environment or may have lacked the necessary kairomone load rate and/or ratio necessary to match the high attractiveness of wild egg masses with their natural kairomones intact. 

Interestingly, the results of the mesocosm environment did not fully align with the field trial results, suggesting lures containing (E)-2-decenel may be repellent, but were better aligned with those of Malek et al. [21] where the 80% n-tridecane ratio was more attractive than n-tridecane alone. The result of wasp attraction to the 100% and 80% lures and unexpected lack of attraction to the 90% treatment occurred both in the field and in the mesocosm trials. Research has suggested that the variable secretions by different sexes and life stages of *H. halys* may be linked to their specific functions at said life stages and physiological states [21,32,33]. Perhaps the 100% and 80% n-tridecane treatments in this study are more closely associated with the gravid/ovipositing adult female or egg life stages that can be exploited by *T. japonicus*, while the 90% treatment is associated with other life stages. Further investigation is necessary to understand these counterintuitive results. 

The mesocosm trials presented the opportunity to test the response of a much larger sample size and demonstrate significant differences between the control and treatments, verifying the validity of these lures. While these trials supported the attractiveness of certain lures in a controlled environment, they also did not include many of the external factors at play in an authentic environment such as plant volatiles and competing parasitoids and predators. In addition, our mesocosm trials did not include the presence of host eggs which may have altered the *T. japonicus* response.

In contrast, the field trials provide essential information about the interaction between hosts and parasitoids in a novel geographic and climatic environment. The results further verify the preliminary research in Utah on the effectiveness of the exotic *T. japonicus* and the common native *T. euschisti*. In this study, as in Holthouse et al. [9], *T. euschisti* demonstrated the ability to parasitize *H. halys* in a similar proportion to the natural parasitoid of *H. halys*, *T. japonicus*. However, *T. euschisti* seems an inviable option for successful biological control due to very low adult wasp emergence and successful stink bug nymph development likely associated with the failure of *T. euschisti* eggs to hatch or early death of larvae within *H. halys* eggs [34]. Additionally, this poses an evolutionary trap for *T. euschisti* and other native *Trissolcus* species that accept *H. halys* eggs as ovipositional sites despite their poor reproductive investment [35]; although, there is evidence that the recent arrival of *T. japonicus* may have implications for the success of native *Trissolcus* on *H. halys* egg masses.

Research has demonstrated that *Trissolcus* spp. are able to parasitize host eggs that have previously been parasitized. In the case of *T. japonicus* and *T. mitsukurri* (Ashmead), each species outperformed the other when it was the first to oviposit, though *T. mitsukurri* was more aggressive in chasing off *T. japonicus* when present concurrently [36]. The similar timing of the competition and outperformance may have been the case in our observation of *T. euschisti* guarding an egg mass (presumable parasitized by *T. japonicus* prior to *T. euschisti* oviposition) that only produced *T. japonicus* offspring. Conversely, research by Konopka et al. [37] demonstrated that parasitism by the exotic *T. japonicus* can provide facultative parasitism opportunities for native *Trissolcus,* such as *T. cultratus* (Mayr), to successfully develop in *H. halys* eggs when they would otherwise fail. 

Regardless of their reproductive success, native *Trissolcus* species can reduce the developmental success of *H. halys* embryos providing low levels of control for the pest [38]. The attraction of *T. eushisti* and other native *Trissolcus* species to kairomone lures containing n-tridecane and (E)-2-decenal were not explored in a mesocosm environment in this study. However, the use of these kairomone lures in the field did result in attacks from *T. euschisti* in addition to *T. japonicus*. Consequently, further research into the physiological and behavioral interactions between the exotic *T. japonicus* and North American native *Trissolcus* requires investigation to fully evaluate the potential efficacy of biological control programs against *H. halys.*

While much investigation is needed into the complex system of parasitoids using semiochemicals to locate their host, the mesocosm results presented support the validity of using rubber septa as a release device for the kairomones of stink bugs, and this research provides not only preliminary results but also a baseline for future work with experimental lures for *H. halys* parasitoids in a field environment.

## 5. Conclusions

As biological control continues to be a preferred approach for managing the invasive *H. halys*, it is important to explore all avenues for attracting and retaining effective parasitoids. Here, we provide support for the validity of infusing rubber septa lures with *H. halys* kairomones to attract *T. japonicus*. This novel strategy has the potential to increase parasitism and, therefore, suppression of *H. halys* in agricultural and urban settings and deserves further investigation.

## Figures and Tables

**Table 1:** Number of *Halyomorpha halys* egg masses parasitized (and percent of eggs with wasp emergence) in kairomone lure treatments for observed parasitoid species. Lure treatments are labeled based on percentage of n-tridecane attractant to (E)-2-decenal repellent; the control contained only hexane. Ninety-two *H. halys* egg masses were deployed containing 2472 eggs on *Catalpa speciosa* leaves in Salt Lake City, UT, USA, from 24 June through 27 August 2021.

	Parasitized Egg Masses (% Emergence)			
Treatment	*T. japonicus*	*T. euschisti*	Unknown	Total
Control	1 (100.0%)	2 (5.5%)	2 (0.0%)	5 (23.0%)
100%	3 (89.0%) *	2 (0.0%)	0 (0.0%)	5 (51.6%)
90%	0 (0.0%)	2 (0.0%)	0 (0.0%)	2 (0.0%)
80%	1 (28.6%)	0 (0.0%)	0 (0.0%)	1 (28.6%)
Total	5 (78.3%)	6 (1.8%)	2 (0.0%)	13 (30.2%)

* A *T. euschisti* female was found guarding an egg mass that resulted in 0% *T. euschisti* and 92.9% *T. japonicus* emergence. It was therefore only counted in the *T. japonicus* column.

## Data Availability

The data presented in this study are available on request from the corresponding author.

## Supplementary Materials

The following supporting information can be downloaded at: https://www.mdpi.com/article/10.3390/insects14020125/s1, Table S1: Statistical results from Kruskal-Wallis tests to compare each egg fate category differences across treatment lure types.
