# Supplementary material for: Efficacy of Kairomone Lures to Attract Parasitoids of Halyomorpha halys
Source: Insects. 2023 Jan 25;14(2):125. doi: 10.3390/insects14020125 (PMC9962316; doi:10.3390/insects14020125)
Supplement: Supplementary file 1 [file insects-14-00125-s001.zip › insects-2165344-supplementary.pdf]

Table S1. Statistical results from Kruskal-Wallis tests to compare each egg fate category differences across treatment lure types.

**Egg Fate**	X^2	*df*	*p*
Hatched *H. halys*	2.79	3	0.426
Missing	2.06	3	0.561
Empty	0.98	3	0.806
Sunken	1.21	3	0.751
Predated	0.22	3	0.974
Unhatched *H. halys*	6.62	3	0.085
Undeveloped parasitoid	5.68	3	0.128
T. euschisti emergence	3.00	3	0.392
T. japonicus emergence	4.01	3	0.260
